# Discrimination of human faces by archerfish (*Toxotes chatareus*)

**DOI:** 10.1038/srep27523

**Published:** 2016-06-07

**Authors:** Cait Newport, Guy Wallis, Yarema Reshitnyk, Ulrike E. Siebeck

**Affiliations:** 1 School of Biomedical Sciences, University of Queensland, Brisbane, Australia; 2 Department of Zoology, University of Oxford, Oxford, England; 3 Centre for Sensorimotor Performance, School of Human Movement Studies, University of Queensland, Brisbane, Australia; 4 ARC Centre of Excellence for Engineered Quantum Systems, School of Mathematics and Physics, University of Queensland, Brisbane, Australia

## Abstract

Two rival theories of how humans recognize faces exist: (i) recognition is innate, relying on specialized neocortical circuitry, and (ii) recognition is a learned expertise, relying on general object recognition pathways. Here, we explore whether animals without a neocortex, can learn to recognize human faces. Human facial recognition has previously been demonstrated for birds, however they are now known to possess neocortex-like structures. Also, with much of the work done in domesticated pigeons, one cannot rule out the possibility that they have developed adaptations for human face recognition. Fish do not appear to possess neocortex-like cells, and given their lack of direct exposure to humans, are unlikely to have evolved any specialized capabilities for human facial recognition. Using a two-alternative forced-choice procedure, we show that archerfish (*Toxotes chatareus*) can learn to discriminate a large number of human face images (Experiment 1, 44 faces), even after controlling for colour, head-shape and brightness (Experiment 2, 18 faces). This study not only demonstrates that archerfish have impressive pattern discrimination abilities, but also provides evidence that a vertebrate lacking a neocortex and without an evolutionary prerogative to discriminate human faces, can nonetheless do so to a high degree of accuracy.

Rapid and accurate recognition of an individual is central to the development of complex social systems that rely on individual identification. Humans are highly adept at this task despite the fact that faces share the same basic components and individuals must be discriminated based on subtle differences in features or spatial relationships^1^,^2^. Considerable evidence points to the fact that the fusiform gyrus, located in the neocortex, is heavily involved in face processing in humans^3^,^4^. While it appears that there is domain specificity for face processing, it is still unknown whether the neurons in the fusiform gyrus are specially evolved for this task^5^, and hence whether faces represent a unique class of object, or if the neurons performing face processing are general object recognition neurons tuned to faces through extensive exposure^6^,^7^,^8^,^9^.

One question we might ask is if there is something special about faces as objects that require specialized recognition circuitry. This can be tested by determining if non-human species that have not evolved to recognize human faces, are nevertheless capable of discriminating human facial stimuli. Evidence that other animals can do this will not unequivocally prove that humans do not use specialized neurons, but it will give an indication if specialized neurons are a requirement and if there is something unusual about faces themselves. There is evidence from a range of studies that some non-primate mammals can discriminate human faces. Species which have been tested include sheep^10^,^11^, dogs^12^,^13^, cows^14^ and horses^15^. However, most animals tested possess a neocortex and have been domesticated, and may, as a result, have experienced evolutionary pressure to recognize their human carers.

There is some evidence that animals lacking a neocortex, namely bees^16^ and birds^17^,^18^,^19^,^20^,^21^,^22^,^23^, are capable of some degree of human facial discrimination. Although the experiments with bees are limited by the small number of test stimuli (a total of four human faces were presented to the bees)^16^, it does indicate that the bee visual recognition system is adequate for a limited version of the task. One reason that human facial recognition is considered a difficult task is that all faces share the same basic features (i.e. two eyes above a nose and a mouth) and therefore discrimination generally requires the ability to detect subtle differences within the set of all faces. The small number of faces presented to the bees makes it possible to discriminate the faces using only trivial visual features, as opposed to the more complex and subtle discrimination required when performing true robust facial discrimination with a large number of faces. Considering that bees and many insects have a much lower visual acuity than most diurnal vertebrates (bees, *Apis mellifera*, for example have a visual acuity of ~0.25 cycles per degree (cpd)^24^ while archerfish have an acuity of ~3.2 cpd^25^ and humans have an acuity of ~72 cpd^26^), it seems questionable that bees would be able to detect enough detail to discriminate more than a small number of faces.

Pigeons (*Columba livia*) can not only discriminate frontal and rotated images of human faces^20^,^27^ but can also categorize them based on expressions^17^,^27^ and gender^18^,^21^. In addition, the performance of pigeons when completing some visual tasks is comparable to that of primates, suggesting that the underlying mechanisms of object recognition, including human facial recognition, may be similar between the two groups^28^,^29^. For example, both humans and pigeons can recognize an individual human face despite some changes in facial expression, while the ability to discriminate emotional expression is associated with a particular face^27^. Chickens (*Gallus gallus domesticus*)^19^ and jungle crows (*Corvus macrorhynchos*)^23^ can also discriminate pictures of human faces, and American crows (*Corvus brachyrhynchos*) recognize individual face masks worn by humans and respond to a particular mask regardless of the person wearing it^22^. This ability may be a result of pre-existing neural specializations as these species often live in urban habitats and interact with humans^30^,^31^ as well as demonstrate conspecific recognition based on visual cues^32^,^33^,^34^,^35^,^36^. We therefore wondered whether teleost fish, the earliest vertebrate taxon lacking neocortical circuitry and one that is unlikely to have evolved any specializations for discriminating human faces, would show similar human face discrimination abilities.

In this report we used archerfish (*Toxotes chatareus*) as a model for behavioural experiments. This species, known for knocking down aerial prey with jets of water, relies heavily on vision to detect small prey against a visually complex background and demonstrates impressive visual cognitive abilities^37^,^38^,^39^,^40^. We hypothesized that this species may be ideally adapted to visual tasks that require sophisticated pattern recognition. Experiments using two stimulus sets were run. In Experiment 1, the fish were presented with 44 colour images of full faces (Fig. 1A). In Experiment 2, the fish were presented with 18 greyscale images of faces that were standardized for brightness (Fig. 1B). In addition, an oval mask was overlaid on the faces to eliminate head shape as a possible recognition cue.

## Results

### Experiment 1

**Figure 1:**
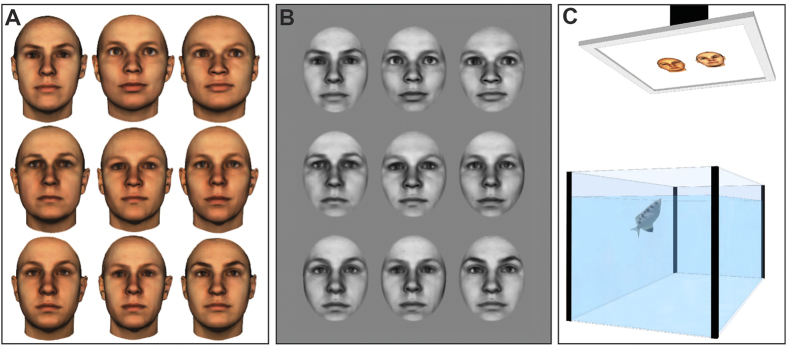
Examples of face images representative of those used in Experiment 1 (**A**) and Experiment 2 (**B**). Images shown are 3D morphs of several faces to protect the privacy of specific individuals. All face images were provided by the Max-Planck Institute for Biological Cybernetics in Tübingen, Germany. (**C**) Illustration of the experimental setup.

**Figure 2:**
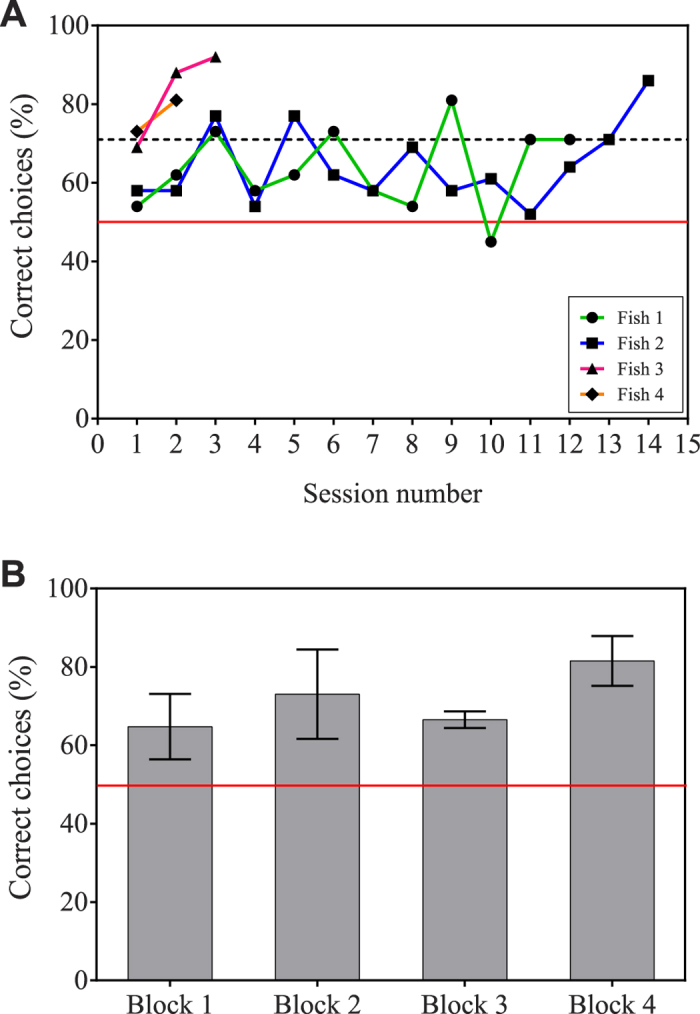
Training and testing results for Experiment 1. (**A**) *Training results.* Fish were trained to select CS+ and avoid CS−. Each curve represents the individual results of a specific fish. The dashed line at 71% represents the training criterion performance level. (**B**) *Testing results.* Fish were trained to avoid CS− and select 44 possible N+. The mean correct selection frequencies for each testing block were calculated. Bars represent standard deviation. The red line at 50% in both figures represents the expected selection frequency if subjects were choosing at random.

In the first experiment, we tested whether four archerfish could be trained using operant conditioning to discriminate between two images of human faces. The images were presented on a computer monitor positioned above the aquarium and the archerfish were required to spit a jet of water at a conditioned stimulus (CS+) and avoid a second conditioned stimulus (CS−) to receive a food reward (Fig. 1C). All fish learned to discriminate between CS+ and CS− within 2–14 sessions (Fig. 2A; Fish 1: 12 sessions; Fish 2: 14 sessions; Fish 3: 3 sessions; Fish 4: 2 sessions).

To determine how robust the discrimination abilities of archerfish are when faced with many faces, we tested whether the fish could continue to discriminate the learned CS− face from 44 novel faces. The task was for fish to continue to avoid the trained distractor (CS−) and select the novel stimuli (N+). Avoidance of CS− rather than selection of CS+ or N+, was used to test the archerfish because Newport, *et al.*^41^ found that archerfish form a stronger association with unrewarded stimuli than with rewarded stimuli. Presentation of the novel faces was divided into two sessions of 29 trials, which were grouped for the purpose of analysis and referred to as a ‘block’. Fish 1 and 4 completed four blocks while Fish 2 and 3 completed two blocks. The individual percentage of correct choices for all four fish was grouped by block and the mean frequency of correct choices and standard deviation for each block was calculated (Block 1: 64.75 ± 8.342; Block 2: 73 ± 11.40; Block 3: 66.5 ± 2.121; Block 4: 81.5 ± 6.364). Our sample size was small (N = 4 fish for each experiment) therefore a Generalized Linear Mixed Model fit by maximum likelihood (Laplace Approximation) with a binomial distribution with logit-link function was used for analysis in both experiments (see Statistical Analysis section for more details). We found the fish were able to discriminate the trained face from the 44 novel ones (*Z* = 5.949, *P* < 0.001, Fig. 2B).

### Experiment 2

**Figure 3:**
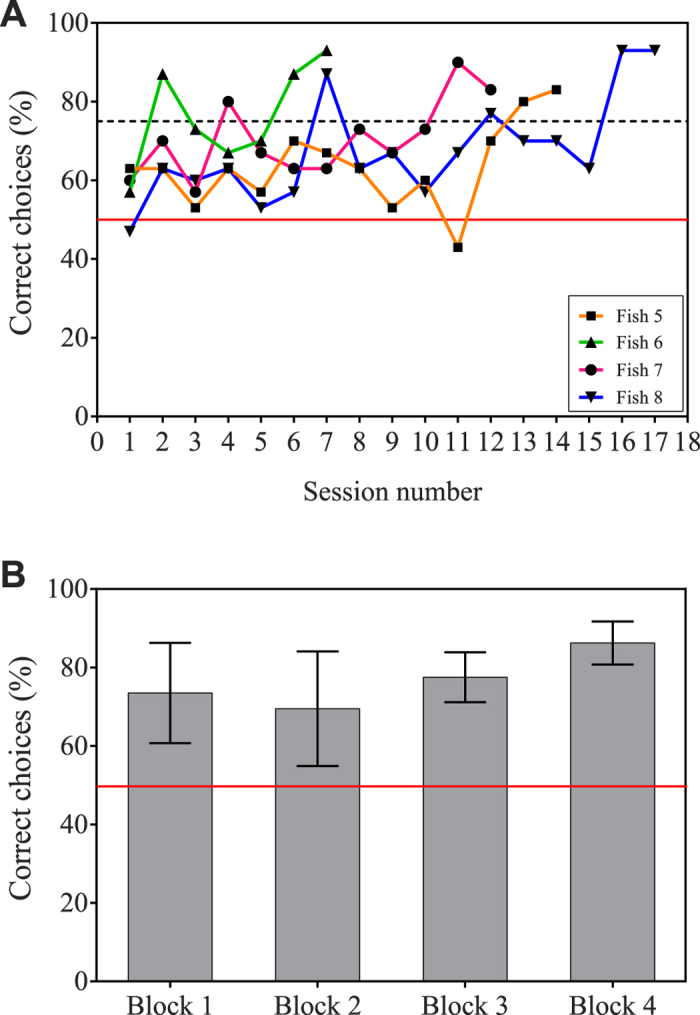
Training and testing results for Experiment 2. (**A**) *Training results.* Fish were trained to select CS+ and avoid CS−. Each curve represents the individual results of a specific fish. The dashed line at 75% represents the training criterion performance level. (**B**) *Testing results.* Fish were trained to avoid CS− and select 18 possible N+. The mean correct selection frequencies for each testing block were calculated. Bars represent standard deviation. The red line at 50% in both figures represents the expected selection frequency if subjects were choosing at random.

In the second experiment, we tested whether four new archerfish could be trained to discriminate human faces when some potentially trivial cues (i.e. brightness, colour and head-shape) were standardized. The general protocol of training and testing was the same as that described for Experiment 1 with the exception that the number of novel stimuli used in testing was 18 and there was no intermediate training step. All fish reached our training criterion within 8–17 sessions (Fig. 3A; Fish 5: 14 sessions; Fish 6: 8 sessions; Fish 7: 7 sessions; Fish 8: 17 sessions). Four testing blocks were then completed by all fish. The individual percentage of correct choices for all four fish was grouped by block and the mean frequency of correct choices and standard deviation for each block were calculated (Block 1: 73.50 ± 12.77; Block 2: 69.50 ± 14.62; Block 3: 77.50 ± 6.351; Block 4: 86.25 ± 5.50). The fish significantly discriminated the trained face from the 18 novel ones (*Z* = 6.794, *P* < 0.001, Fig. 2B).

## Discussion

We tested whether a species of fish, unlikely to have experienced any evolutionary pressure for human facial recognition, could learn to discriminate human faces. We found that archerfish could be trained to discriminate a learned face from a large number of other human faces even when some trivial cues had been removed (i.e. brightness, colour and head-shape). While it is impossible to say from our study whether archerfish use the same visual information to discriminate the face images as humans, our results clearly show that some aspects of the facial recognition task can be learnt, even in the absence of a neocortex.

During testing in Experiments 1 and 2, all fish reached peak discrimination accuracy between 77–89%. Archerfish have previously been shown capable of discriminating large numbers of stimuli to a similar degree of accuracy (up to 93% accuracy)^41^,^42^. It seems likely that the archerfish did not use trivial features to discriminate the human faces as the fish could distinguish one face from 44 others which varied in similarity. In addition, when brightness, colour and general outline cues were standardized, the fish were still able to complete the task. Our results demonstrate that, like some species of reef fish^43^, archerfish are adept at fine-detail pattern discrimination and can apply these abilities to unfamiliar stimuli, including human faces.

During training, we observed individual variation in the number of sessions required to learn the task; while some fish learned within a single session (Experiment 1: Fish 3 and 4), others required longer periods of training (up to 17 sessions / 510 trials). The difference in learning rates may simply be due to individual factors such as experience and motivation. However, it is also possible that individuals used different visual information to discriminate the faces and that some features required more time to learn. If individuals do learn to use different visual information for discrimination, it may also explain why some fish achieved a higher degree of accuracy than others in the testing period. When it comes to visually identifying an object, not all visual information is created equal. For example, by learning the combined appearance of the eyes, nose and mouth of a particular human face, it is likely you will be able to easily identify that face from a large pool of other faces. However, learning the appearance of a single spot on the cheek is not likely to be as helpful.

During testing, we saw a similar pattern of behavior. Some fish were immediately highly accurate (Experiment 1: Fish 3 and 4; Experiment 2: Fish 5, 7 and 8), while others improved with experience (Experiment 1: Fish 1 and 2; Experiment 2: Fish 6). These differences in individual performance provide additional evidence that some of the fish were using different features for facial identification from the others and that this visual information differed in its effectiveness for the discrimination task. Future experiments testing which features the fish use to discriminate faces would help shed light on whether individual fish use different features and if these feature were similar to those used by human observers. There are several experimental methods that involve altering facial stimuli in some way (e.g.^44^,^45^,^46^,^47^) which have previously been used to explore feature use by primates^44^,^45^,^46^,^47^ and pigeons^21^ when discriminating human faces and these approaches may be adaptable for future studies with fish.

Understanding the recognition capabilities of different animals can inform us about the evolutionary history of human facial recognition. There are a wide range of animals that use visual cues for conspecific individual recognition including primates e.g.^47^,^48^, crayfish^49^, fiddler crabs^50^, sheep^51^,^52^, damselfish^43^ and wasps^53^. With so many examples across such diverse taxa, it is clear that the discrimination of individuals based on facial features is not unique to humans and suggests that perhaps human faces themselves are not a particularly special class of objects. Our evidence that archerfish can discriminate human faces without having any obvious selection pressure for this specific task, suggests that the visual system of distantly related vertebrates is capable of sophisticated discrimination tasks. This is not surprising as so many behaviours fundamental to the survival of a wide range of species rely on accurate vision-based object recognition, including predator detection, mate selection, and feeding. Therefore it seems possible that pre-existing circuits for sophisticated visual discrimination evolved into the dedicated face-processing circuitry of primates.

In this experiment we tested discrimination of frontal views; this is a very restricted version of the task humans must perform in order to rapidly and accurately discriminate human faces in real situations. Faces are dynamic and their appearance can be drastically changed by a range of factors including variations in viewing angle, lighting, or facial expression. Unlike the faces of many other vertebrates, primate faces have complex musculature allowing them to form a broad range of facial expressions^2^. It is possible that the complexity of the neocortex is a requirement for the discrimination of faces under variable conditions. That said, there is evidence that pigeons are able to recognize faces that have changed in viewing angle^20^ and expression^17^. This has yet to be tested in animals such as fish that do not live near humans, however, many social animals that recognize conspecific individuals are equally capable of discriminating those individuals under a range of viewing conditions. Fish present an interesting example as they can use colour patterns for recognition which are additionally affected by changes in water quality and lighting. Because different wavelengths are attenuated unequally in water, some colours within a pattern are affected more than others. It is possible that the perceived complexity of human facial recognition may simply be an anthropogenic point of view and in fact other animals must also perform similarly complex pattern discrimination tasks under highly demanding conditions^43^.

## Methods

### Subjects

The archerfish used in this experiment were kept as described in Newport, *et al.*^41^. All fish were kept in accordance with the University of Queensland Animal Ethics Committee approval (AEC approval number: SBMS/241/12) and all experimental protocols were approved by the same body. The fish had different levels of previous experience, however all subjects had at least been pre-trained to spit at stimuli presented on a computer monitor, following methods described in Newport, *et al.*^41^.

### Stimuli

The images used were acquired from a database of three-dimensional head models created by researchers at the Max Planck Institute in Tübingen, Germany^54^,^55^,^56^. A total of 62 frontal views of Caucasian female human faces (rendered size: 384 × 384 pixels) were used as stimuli (see examples in Fig. 1A,B). The images in this database have had extraneous cues (e.g. hair and clothing) removed thereby reducing the possibility that trivial features could be used to discriminate the faces.

Three aspects of the images in Experiment 2 were standardized. 1) An identical oval mask was overlaid on all images to remove head-shape as a possible cue. 2) The images were then converted to grayscale, where pixels can have individual values between 0–255, to remove colour cues. 3) The brightness of each image was normalized so that all images had a mean brightness of 128.

### General procedure

The experimental apparatus and stimulus presentation were as described in Newport, *et al.*^41^. Briefly, stimuli were presented on an LCD monitor (1024 × 768 pixels) suspended above the aquaria and oriented parallel to the water’s surface. A two-alternative forced choice (2-AFC) procedure was used and images were displayed on each half of the monitor (monitor coordinates: 0–160, 0 160), one of which was rewarded if hit. Fish were rewarded with one food pellet (Cichlid Gold, Kyorin Co. Ltd., Japan) each time they selected the correct stimulus by hitting it with a jet of water. Selection of the incorrect stimulus terminated the trial.

### Training and testing for Experiment 1

Archerfish were trained to discriminate between one rewarded face (CS+) and one unrewarded face (CS−). Fish 1 & 2 and Fish 3 & 4 were trained with opposite faces as CS− to reduce the possibility that performance was due to a unique characteristic of a particular face. Each training session consisted of 21–31 trials, depending on the individual level of motivation in a particular session. Training sessions were repeated until the subjects had achieved a statistically significant correct choice frequency of ≥71% (binomial test: P < 0.05, N = 21 trials) in two consecutive sessions.

In an intermediary training step, the fish learned to discriminate CS− from eight novel face images (N+). For each trial, one N+ was chosen randomly from the pool of stimuli with the constraint that the same N+ was not used in consecutive trials and that the presentation of all eight stimuli was balanced within a session. One trial with the original CS+ and CS− was included in the pool as task reinforcement. Sessions were run until the fish reached our training criterion (correct choice frequency of ≥71% in two consecutive sessions).

During testing, a pool of 44 faces was used as novel stimuli. Within a block, 10 reinforcement trials (CS+ and CS−) and four of the faces used during the intermediary training step were used as training reminders. Trials with previously seen stimuli were excluded from analysis, therefore a testing block consisted of 44 trials.

### Training and testing for Experiment 2

Training was run generally using the same procedures as in Experiment 1, however, no intermediary training stage was used as we felt it had little impact on the ability of the fish to complete the task. All fish were trained to different faces as CS− to reduce the possibility that performance was due to a unique characteristic of a particular face. Each training session consisted of 30 trials and sessions were repeated until the subjects had achieved a correct choice frequency of ≥75% (binomial test: P < 0.01, N = 30 trials) in two consecutive sessions.

During testing, a pool of 18 novel faces was used. Within each block, one reinforcement trial (the original CS+ and CS−) was run but was excluded from analysis, therefore a testing block consisted of 18 trials. A single session consisted of 30 trials, therefore more than one block was completed per session.

### Statistical analyses

For each experiment we used a Generalized Linear Mixed Model with a binomial distribution with log-link function. A binary outcome (correct/incorrect) was used as the dependent variable. Fish ID and block number were included as random factors. In addition, Fish ID and block number were included as separate, crossed random factors. Variance due to individual fish choices in both experiments was small (<0.01), indicating that the fish made similar choices.

## Additional Information

**How to cite this article**: Newport, C. *et al.* Discrimination of human faces by archerfish (*Toxotes chatareus*). *Sci. Rep.* **6**, 27523; doi: 10.1038/srep27523 (2016).
